# Crystal structure of bis­(2,2′-bi­pyridine)[*N*′-(quino­lin-2-ylmethylidene)pyridine-2-carbohydrazide]ruthenium(II) bis(tetra­fluorido­borate) di­chloro­methane tris­olvate

**DOI:** 10.1107/S2056989015000122

**Published:** 2015-01-10

**Authors:** Asami Mori, Takayoshi Suzuki, Kiyohiko Nakajima

**Affiliations:** a Department of Chemistry, Faculty of Science, Okayama University, Okayama, 700-8530, Japan; b Department of Chemistry, Aichi University of Education, Kariya, Aichi 448-8542, Japan

**Keywords:** crystal structure, picolinolylhydrazone, intra­molecular hydrogen bonding, ruthenium(II) polypyridyl complex.

## Abstract

The title compound, [Ru(C_10_H_8_N_2_)_2_(C_16_H_12_N_4_O)](BF_4_)_2_·3CH_2_Cl_2_, crystallizes with one complex dication, two BF_4_^−^ counter-anions and three di­chloro­methane solvent mol­ecules in the asymmetric unit. The central Ru^II^ atom adopts a distorted octa­hedral coordination sphere with two 2,2′-bi­pyridine (bpy) and one quinoline-2-carbaldehyde (pyridine-2-carbon­yl)hydrazone (H*L*) ligand. The hydrazone ligand has a *Z* form and coordinates to the Ru^II^ atom *via* the amide-O and imine-N atoms, affording a planar five-membered chelate ring, while its pyridine-N and quinoline-N donor atoms in the substituents are non-coordinating. The hydrazone N—H group forms an intra­molecular hydrogen bond with the quinoline-N atom. In the crystal, the quinoline moiety of H*L* shows the shortest π–π stacking inter­action with the pyridine substituent of H*L* in a neighbouring complex, the centroid-to-centroid distance being 3.793 (3) Å.

## Abstract 2

2-Picolinoylhydrazone with a 2-quinolyl substituent on the imine-C atom coordinates in the neutral *Z* form to a Ru^II^(bpy)_2_ fragment *via* the amide-O and imine-N atoms, affording a planar five-membered chelate ring, and its hydrazone N—H group forms an intra­molecular hydrogen bond with the uncoordinating quinoline-N atom.

## Chemical context   

Aroylhydrazones, *Ar*C(O)NHN=CH*R*, are easily prepared by the reaction of an aroylhydrazine [*Ar*C(O)NHNH_2_] with an aldehyde (*R*CHO), and they can coordinate to a metal atom *via* the amide-O and imine-N atoms (Bernhardt *et al.*, 2007 ▸; Raveendran & Pal, 2005 ▸, 2006 ▸). These hydrazones are often obtained as a mixture of *E* and *Z* isomers (Su & Aprahamian, 2014 ▸), and both isomers are generally weak acids. However, when they coordinate to a metal ion through the imine-N atom, their acidity becomes higher (Chang *et al.*, 2010 ▸), and the deprotonated hydrazonato complexes are often isolated (Nonoyama, 1974 ▸). For example, the reaction of *cis*-[RuCl_2_(bpy)_2_] (bpy is 2,2′-bi­pyridine) and a series of aroylhydrazones in the presence of tri­ethyl­amine afforded the cationic complexes [Ru^II^(bpy)_2_(hydrazonato)](ClO_4_ or PF_6_), which were unambiguously characterized by X-ray analysis (Duan *et al.*, 1998 ▸; Ghosh *et al.*, 2014 ▸).

In the current study we utilized a 2-picolinoylhydrazone (*Ar* = 2-C_5_H_4_N) with a 2-quinolyl substituent on the imine-C atom (*R* = 2-C_9_H_6_N). This compound (H*L*) has several possible coordination modes because of the additional pyridine and quinoline ligating groups. In a previous study we investigated the reaction products from [RuCl_2_(PPh_3_)_3_] and (an *E*/*Z* mixture of) H*L* under several reaction conditions, and characterized three geometrical isomers of [RuCl_2_(PPh_3_)_2_{H*L*-κ*O*(amide),κ*N*(imine)}] as well as a linkage isomer of *trans*(P)-[RuCl_2_(PPh_3_)_2_{H*L*-κ*N*(imine), κ*N*(quinoline)}] (Mori *et al.*, 2014 ▸). Here, we have examined the reaction of the *Z* isomer of H*L* and an Ru^II^(bpy)_2_ precursor prepared from *cis*-[RuCl_2_(bpy)_2_] and AgBF_4_ (2 eq.) in ethanol. The resulting orange product had the composition Ru(bpy)_2_(H*L*)(BF_4_)_2_, indicating the formation of an Ru^II^ complex with a neutral hydrazone ligand, in contrast to the previous examples of [Ru^II^(bpy)_2_(hydrazonato)](ClO_4_ or PF_6_). Therefore, in order to determine the mol­ecular and crystal structure of the present product, an orange prismatic crystal of the title compound, (I), [Ru(C_10_H_8_N_2_)_2_(C_16_H_12_N_4_O)](BF_4_)_2_·3CH_2_Cl_2_, was analysed by X-ray diffraction.
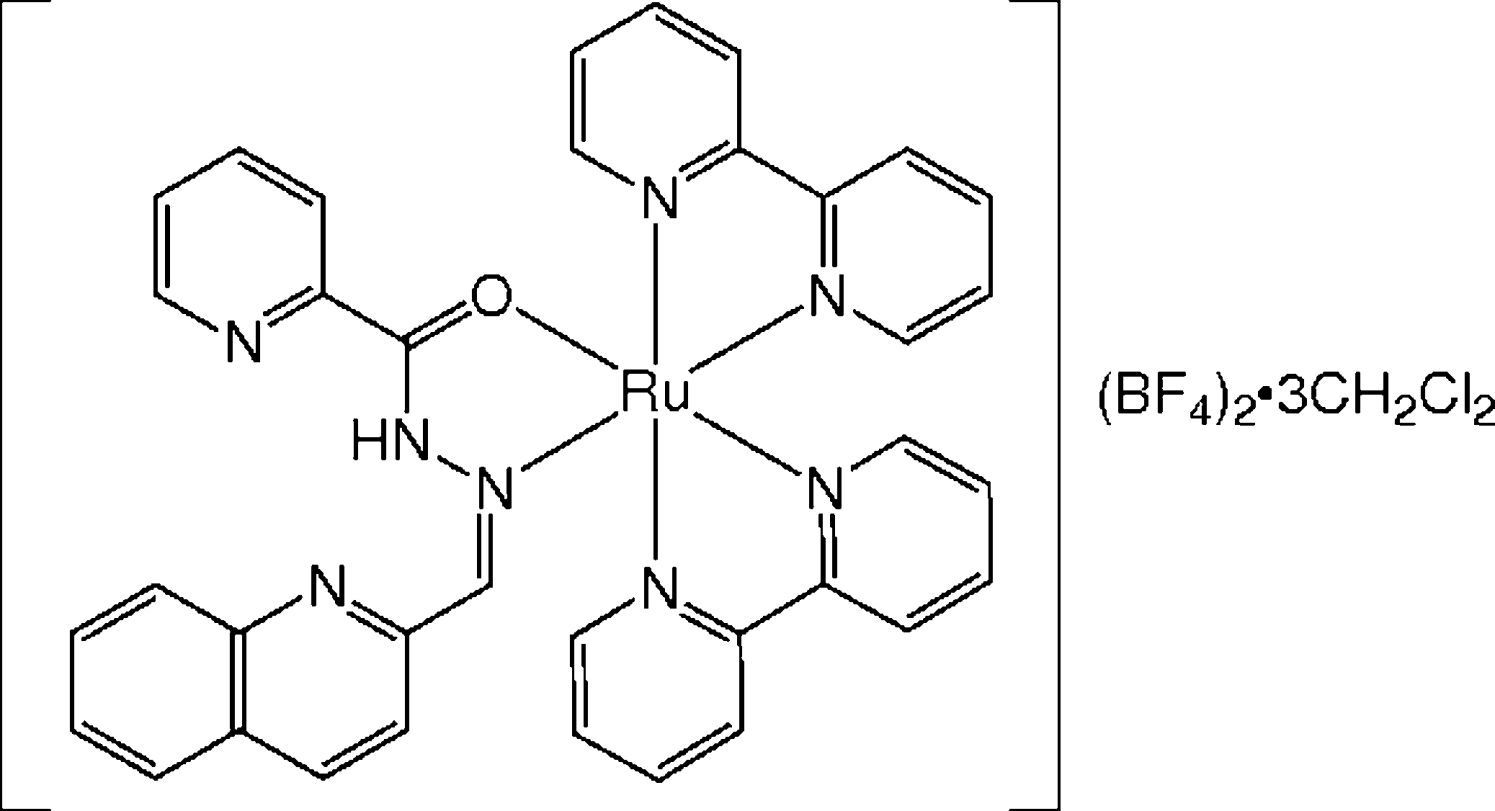


## Structural commentary   

**Figure 1:**
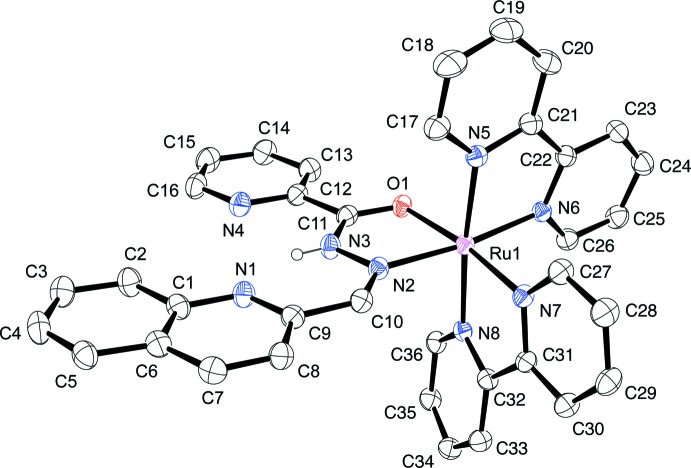
View of the mol­ecular structure of the cationic complex in the title compound, showing the atom-numbering scheme, with displacement ellipsoids drawn at the 30% probability level. Hydrogen atoms except for the hydrazone N—H group are omitted for clarity.

The asymmetric unit of compound (I) contains one complex dication (Fig. 1 ▸), two BF_4_^−^ counter-anions and three di­chloro­methane solvent mol­ecules. In the cationic complex, the neutral hydrazone is present as its *Z* isomer and coordinates to the Ru^II^ atom through the amide-O and imine-N atoms, forming a virtually planar five-membered chelate ring [maximum deviation from the least-squares plane = 0.015 (4) Å], as well as two bidentate bpy co-ligands. An intra­molecular hydrogen bond between the hydrazone N—H group and the quinoline-N atom is observed (Table 1 ▸). The pyridine (py) and quinoline (qn) moieties of H*L* are non-coordinating, but their mean planes are almost co-planar to the Ru^II^ carb­oxy­lic acid hydrazide (CAH: —C(O)NHN=) chelating plane. The dihedral angles between these planes are: py *vs* CAH = 5.4 (2), qn *vs* CAH = 3.7 (2) and py *vs* qn = 2.3 (2)°.

The Ru1—O1(amide) and Ru1—N2(imine) bond lengths in (I) are 2.090 (3) and 2.047 (4) Å, respectively, which are comparable to those in [Ru(bpy)_2_{3-py-C(O)NN=CHC_6_H_4_(4-NMe_2_)}]ClO_4_ [2.083 (1) and 2.040 (1) Å, respectively; Duan *et al.*, 1998 ▸] and [Ru(bpy)_2_{2-C_6_H_4_(OH)–C(O)NN=CH-2-fur­yl}]PF_6_ [2.072 (2) and 2.089 (1) Å, respectively; Ghosh *et al.*, 2014 ▸]. The bite angle of the hydrazone chelate, O1—Ru—N2, in (I) is 77.8 (1)°, which is also similar to the above-mentioned hydrazonato complexes, 78.0 (1) and 78.6 (1)°, respectively. Thus, the substituent groups on the carbonyl-C and the imine-C atoms of the aroylhydrazones, as well as the protonation (or deprotonation) states of the hydrazone N—H moiety, do not significantly affect the structural parameters of the Ru^II^—(hydrazone/hydrazonate) coordination bonds.

## Supra­molecular features   

**Figure 2:**
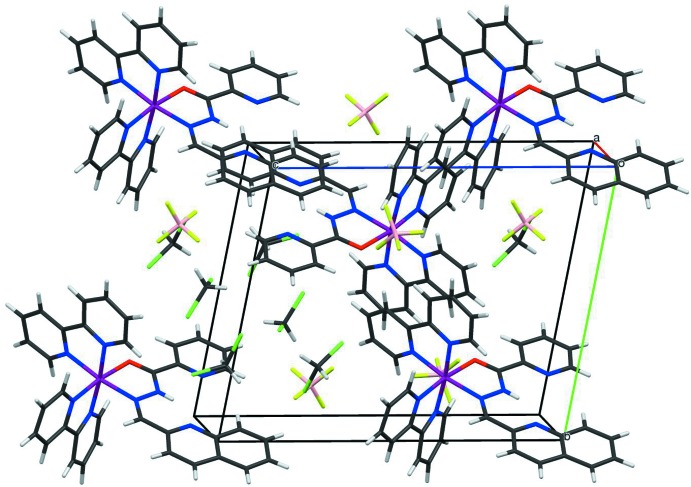
View of the crystal packing of the title compound, illustrating three π–π stacking inter­actions between the complex cations. Colour code: Ru, purple; Cl, green; F, yellow–green; O, red; N, blue; C, black; B, pink; H, grey.

In the crystal structure of (I) there are no remarkable hydrogen-bonding inter­actions between the cationic complex, BF_4_^−^ anions and the solvated di­chloro­methane mol­ecules. However, each of the planar H*L* and two bpy ligands in the complex cation shows a π–π stacking inter­action with the respective neighbouring complexes (Fig. 2 ▸). The quinoline plane (N1/C1–C9) has a stacking inter­action with the pyridine plane (N4^i^/C12^i^–C16^i^) of H*L* in a neighbouring complex [symmetry code: (i) –*x*, –*y* + 1, –*z* + 2]; the shortest C⋯C distance between these rings is C6⋯C16^i^ = 3.444 (8) Å and the centroid-to-centroid distance between the planes C1–C6 and N4^i^/C12^i^–C16^i^ is 3.793 (3) Å. One of the bpy ligands, N5/C17–C26/N6, is stacked with the same symmetry-related ligand, N5^ii^/C17^ii^–C26^ii^/N6^ii^, in a neighbouring complex [symmetry code: (ii) –*x*, –*y* + 1, –*z* + 1]; the shortest C⋯C distance between them is C20⋯C25^i^ = 3.373 (8) Å, and the centroid-to-centroid distance between the N6/C22–C26 and N6^ii^/C22^ii^–C26^ii^ planes is 3.864 (3) Å. For the other bpy ligand, N7/C27–C36/N8, a similar inter­action is observed, and the shortest C⋯C distance between them is C32⋯C35^iii^ = 3.509 (8) Å and the centroid-to-centroid distance between planes N8/C32–C36 and N8^iii^/C32^iii^–C36^iii^ is 3.918 (3) Å [symmetry code: (iii) –*x*, –*y*, –*z* + 1]. Considering these stacking inter­actions, the complex cations are arranged in a three-dimensional extended structure in the crystal.

## Database survey   

Four geometrical and linkage isomers of [RuCl_2_(PPh_3_)_2_(H*L*)] with the same picolinoylhydrazone ligand, H*L*, have been reported previously (Mori *et al.*, 2014 ▸). There is no record of any [Ru^II^(bpy)_2_(carbonyl­hydrazone)]^2+^ complexes with its protonated (neutral) hydrazone form in the CSD database (Version 5.35, last update May 2014; Groom & Allen, 2014 ▸). The deprotonated (anionic) hydrazonate analogues, [Ru(bpy)_2_{3-py-C(O)NN=CHC_6_H_4_(4-NMe_2_)}]ClO_4_ (Duan *et al.*, 1998 ▸) and [Ru(bpy)_2_{2-C_6_H_4_(OH)–C(O)NN=CH-2-fur­yl}]PF_6_ as well as the thio­phene analogue have been reported (Ghosh *et al.*, 2014 ▸). The structurally related compound [Ru^II^(bpy)_2_{C_6_H_5_C(O)NNC_6_H_5_}]PF_6_ with a monoanionic (radical) ligand has also been reported (Ehret *et al.*, 2012 ▸).

## Synthesis and crystallization   

All reagents and solvents were commercially available and used without further purification. The starting ruthenium(II) complex, *cis-*[RuCl_2_(bpy)_2_]·2H_2_O (Sullivan *et al.*, 1978 ▸), and hydrazone ligand, *Z-*H*L* (Mori *et al.*, 2014 ▸), were prepared according to literature procedures. A mixture of *cis-*[RuCl_2_(bpy)_2_]·2H_2_O (618 mg, 1.19 mmol) and AgBF_4_ (463 mg, 2.38 mmol) in ethanol (80 ml) was stirred in the dark at room temperature overnight. The resulting white precipitate (AgCl) was filtered off, and *Z-*H*L* (328 mg, 1.19 mmol) was added to the filtrate. The mixture was heated to reflux for 9 h and then cooled to room temperature. The solution was concentrated to *ca*. 10 ml under reduced pressure, and the resulting microcrystalline powder was collected by filtration and dried in air. Yield: 869 mg (81%). Analysis calculated for C_36_H_28_B_2_F_8_N_8_ORu·2H_2_O: C 48.08, H 3.59, N 12.46%. Found: C 48.11, H 3.42, N 12.18%. Orange prismatic crystals of (I) suitable for X-ray analysis were obtained by diffusion of layered hexane into a di­chloro­methane solution.

## Refinement   

Crystal data, data collection and structure refinement details are summarized in Table 2 ▸. The position of the hydrazone (N—)H atom was located in a difference Fourier map and refined with *U*_iso_ = 1.2*U*_eq_(N). All other H atoms were refined using a riding model, with C—H = 0.95 (aromatic) or 0.99 (methyl­ene) Å and *U*_iso_ = 1.2*U*_eq_(C).

## Supplementary Material

Crystal structure: contains datablock(s) I. DOI: 10.1107/S2056989015000122/wm5107sup1.cif

Structure factors: contains datablock(s) I. DOI: 10.1107/S2056989015000122/wm5107Isup2.hkl

CCDC reference: 1042209

Additional supporting information:  crystallographic information; 3D view; checkCIF report

## Figures and Tables

**Table 1:** Hydrogen-bond geometry (, )

*D*H*A*	*D*H	H*A*	*D* *A*	*D*H*A*
N3H1N1	0.76(6)	1.90(6)	2.553(6)	145(6)

**Table 2:** Experimental details

Crystal data	
Chemical formula	[Ru(C_10_H_8_N_2_)_2_(C_16_H_12_N_2_)](BF_4_)_2_3CH_2_Cl_2_
*M*_r_	1118.13
Crystal system, space group	Triclinic, *P* 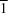
Temperature (K)	192
*a*, *b*, *c* ()	11.0165(12), 13.2508(15), 16.4285(19)
, , ()	77.812(4), 76.924(4), 88.367(4)
*V* (^3^)	2282.9(4)
*Z*	2
Radiation type	Mo *K*
(mm^1^)	0.77
Crystal size (mm)	0.40 0.30 0.25

Data collection	
Diffractometer	Rigaku R-AXIS RAPID
Absorption correction	Numerical (*NUMABS*; Rigaku, 1999 ▸)
*T*_min_, *T*_max_	0.658, 0.825
No. of measured, independent and observed [*I* > 2(*I*)] reflections	22472, 10378, 8147
*R*_int_	0.076
(sin /)_max_ (^1^)	0.649

Refinement	
*R*[*F* ^2^ > 2(*F* ^2^)], *wR*(*F* ^2^), *S*	0.069, 0.208, 1.04
No. of reflections	10378
No. of parameters	589
H-atom treatment	H atoms treated by a mixture of independent and constrained refinement
_max_, _min_ (e ^3^)	1.49, 1.02

Computer programs: *RAPID-AUTO* (Rigaku, 2006 ▸), *CrystalStructure* (Rigaku, 2010 ▸), *SIR2004* (Burla *et al.*, 2005 ▸), *SHELXL2013* (Sheldrick, 2008 ▸) and *ORTEP-3 for Windows* (Farrugia, 2012 ▸).

## supplementary crystallographic information

### Crystal data

**Table:**

[Ru(C_10_H_8_N_2_)_2_(C_16_H_12_N_2_)](BF_4_)_2_·3CH_2_Cl_2_	*Z* = 2
*M**_r_* = 1118.13	*F*(000) = 1120
Triclinic, *P*1	*D*_x_ = 1.627 Mg m^−^^3^
*a* = 11.0165 (12) Å	Mo *K*α radiation, λ = 0.71075 Å
*b* = 13.2508 (15) Å	Cell parameters from 15690 reflections
*c* = 16.4285 (19) Å	θ = 3.0–27.6°
α = 77.812 (4)°	µ = 0.77 mm^−^^1^
β = 76.924 (4)°	*T* = 192 K
γ = 88.367 (4)°	Prism, orange
*V* = 2282.9 (4) Å^3^	0.40 × 0.30 × 0.25 mm

### Data collection

**Table:**

Rigaku R-AXIS RAPID diffractometer	8147 reflections with *I* > 2σ(*I*)
Detector resolution: 10.000 pixels mm^-1^	*R*_int_ = 0.076
ω scans	θ_max_ = 27.5°, θ_min_ = 3.0°
Absorption correction: numerical (*NUMABS*; Rigaku, 1999)	*h* = −14→13
*T*_min_ = 0.658, *T*_max_ = 0.825	*k* = −16→17
22472 measured reflections	*l* = −21→21
10378 independent reflections	

### Refinement

**Table:**

Refinement on *F*^2^	Primary atom site location: structure-invariant direct methods
Least-squares matrix: full	Secondary atom site location: difference Fourier map
*R*[*F*^2^ > 2σ(*F*^2^)] = 0.069	Hydrogen site location: inferred from neighbouring sites
*wR*(*F*^2^) = 0.208	H atoms treated by a mixture of independent and constrained refinement
*S* = 1.04	*w* = 1/[σ^2^(*F*_o_^2^) + (0.1064*P*)^2^ + 2.2063*P*] where *P* = (*F*_o_^2^ + 2*F*_c_^2^)/3
10378 reflections	(Δ/σ)_max_ = 0.001
589 parameters	Δρ_max_ = 1.49 e Å^−^^3^
0 restraints	Δρ_min_ = −1.02 e Å^−^^3^

### Special details

**Table:**

Experimental. ^1^H NMR (600 MHz, 22 °C, CD_3_CN): δ = 9.08 (d, *J* = 4.5 Hz, 1H), 8.84 (d, *J* = 5.6 Hz, 1H), 8.61 (d, *J* = 5.6 Hz, 1H), 8.58 (d, *J* = 8.2 Hz, 2H), 8.56 (d, *J* = 8.7 Hz, 1H), 8.52 (d, *J* = 8.1 Hz, 1H), 8.48 (d, *J* = 7.8 Hz, 1H), 8.48 (d, *J* = 8.6 Hz, 1H), 8.19 (td, *J* = 5.4, 7.6 Hz, 2H), 8.13 (d, *J* = 7.8 Hz, 1H), 8.08–8.04 (m, 4H), 8.01 (td, *J* = 5.3, 1.3 Hz, 1H), 7.83 (t, *J* = 7.6 Hz, 1H), 7.79–7.77 (m, 2H), 7.74 (d, *J* = 6.4 Hz, 1H), 7.65 (ddd, *J* = 5.0, 3.9, 1.4 Hz, 1H), 7.60 (ddd, *J* = 5.0, 3.9, 1.4 Hz, 1H), 7.58 (d, *J* = 8.7 Hz, 1H), 7.46 (s, azomethine-H, 1H), 7.39 (ddd, *J* = 5.0, 4.0, 1.3 Hz, 1H), 7.33 (ddd, *J* = 4.5, 3.9, 1.5 Hz, 1H) p.p.m.. UV-vis (in CH_3_CN) {*λ*_max_/nm (*ε*_max_*M*^-1^ cm^-1^)}: 488 (16600), 332 (18000), 287 (62200), 237 (36700). Cyclic voltammetry (CH_3_CN with 0.1 *M* Bu_4_NClO_4_) {*E*_1/2_/V *vs*. Fc^+^/Fc (Δ*E*/mV) assignment}: 0.79 (80) Ru^III^/Ru^II^, –1.04 (72) bpy/bpy^–^, –1.64 (69) bpy/bpy^–^.
Geometry. All e.s.d.'s (except the e.s.d. in the dihedral angle between two l.s. planes) are estimated using the full covariance matrix. The cell e.s.d.'s are taken into account individually in the estimation of e.s.d.'s in distances, angles and torsion angles; correlations between e.s.d.'s in cell parameters are only used when they are defined by crystal symmetry. An approximate (isotropic) treatment of cell e.s.d.'s is used for estimating e.s.d.'s involving l.s. planes.

### Fractional atomic coordinates and isotropic or equivalent isotropic displacement parameters (Å^2^)

**Table:**

	*x*	*y*	*z*	*U*_iso_*/*U*_eq_	
Ru1	0.13367 (3)	0.24405 (3)	0.60979 (2)	0.03128 (14)	
Cl1	0.3771 (2)	0.4243 (2)	0.9729 (2)	0.1164 (9)	
Cl2	0.4195 (2)	0.28556 (19)	0.85446 (17)	0.1000 (7)	
Cl3	−0.0761 (3)	0.5854 (4)	0.8926 (3)	0.1842 (19)	
Cl4	0.1269 (3)	0.4703 (3)	0.8292 (2)	0.1411 (12)	
Cl5	0.3573 (4)	0.3917 (3)	0.2711 (2)	0.1552 (15)	
Cl6	0.3391 (3)	0.2153 (3)	0.1932 (3)	0.1427 (12)	
F1	0.5184 (4)	0.2510 (4)	0.6277 (3)	0.0938 (14)	
F2	0.6920 (4)	0.2642 (3)	0.6752 (3)	0.0857 (13)	
F3	0.6154 (4)	0.1074 (3)	0.6818 (3)	0.0758 (11)	
F4	0.7026 (4)	0.2090 (3)	0.5556 (2)	0.0735 (10)	
F5	0.7015 (4)	0.2350 (3)	0.1999 (3)	0.0807 (12)	
F6	0.7975 (6)	0.2465 (3)	0.3049 (3)	0.0990 (16)	
F7	0.7015 (3)	0.0997 (3)	0.3094 (2)	0.0667 (9)	
F8	0.8746 (4)	0.1479 (5)	0.2093 (3)	0.1044 (17)	
O1	−0.0113 (3)	0.2934 (2)	0.69791 (18)	0.0355 (6)	
N1	0.1994 (4)	0.0784 (3)	0.8936 (2)	0.0405 (9)	
N2	0.1737 (4)	0.1702 (3)	0.7231 (2)	0.0346 (8)	
N3	0.0834 (4)	0.1913 (3)	0.7909 (2)	0.0391 (9)	
N4	−0.0749 (4)	0.2339 (3)	0.9244 (3)	0.0455 (9)	
N5	0.2334 (3)	0.3775 (3)	0.5961 (2)	0.0339 (8)	
N6	0.0848 (3)	0.3335 (3)	0.5041 (2)	0.0334 (7)	
N7	0.2688 (3)	0.1771 (3)	0.5329 (2)	0.0341 (8)	
N8	0.0439 (3)	0.1104 (3)	0.6106 (2)	0.0317 (7)	
C1	0.2168 (5)	0.0357 (4)	0.9730 (3)	0.0412 (10)	
C2	0.1309 (5)	0.0572 (4)	1.0445 (3)	0.0499 (12)	
H2	0.0612	0.0990	1.0369	0.060*	
C3	0.1478 (6)	0.0181 (5)	1.1250 (3)	0.0555 (14)	
H3	0.0900	0.0328	1.1733	0.067*	
C4	0.2513 (6)	−0.0441 (5)	1.1362 (3)	0.0542 (13)	
H4	0.2632	−0.0701	1.1923	0.065*	
C5	0.3334 (5)	−0.0673 (4)	1.0690 (3)	0.0464 (11)	
H5	0.4007	−0.1114	1.0783	0.056*	
C6	0.3207 (5)	−0.0268 (4)	0.9845 (3)	0.0419 (10)	
C7	0.4028 (5)	−0.0460 (4)	0.9110 (3)	0.0445 (11)	
H7	0.4717	−0.0896	0.9164	0.053*	
C8	0.3847 (5)	−0.0026 (4)	0.8321 (3)	0.0420 (10)	
H8	0.4413	−0.0143	0.7822	0.050*	
C9	0.2804 (4)	0.0601 (4)	0.8257 (3)	0.0380 (9)	
C10	0.2615 (4)	0.1096 (3)	0.7413 (3)	0.0363 (9)	
H10	0.3216	0.0952	0.6939	0.044*	
C11	−0.0066 (4)	0.2541 (4)	0.7732 (3)	0.0362 (9)	
C12	−0.0996 (4)	0.2757 (4)	0.8476 (3)	0.0383 (10)	
C13	−0.2028 (5)	0.3326 (4)	0.8370 (3)	0.0449 (11)	
H13	−0.2171	0.3587	0.7815	0.054*	
C14	−0.2851 (5)	0.3510 (5)	0.9086 (4)	0.0523 (12)	
H14	−0.3570	0.3911	0.9039	0.063*	
C15	−0.2605 (5)	0.3099 (5)	0.9873 (4)	0.0546 (13)	
H15	−0.3157	0.3214	1.0378	0.066*	
C16	−0.1564 (5)	0.2522 (4)	0.9926 (3)	0.0508 (12)	
H16	−0.1416	0.2240	1.0476	0.061*	
C17	0.3043 (5)	0.3965 (4)	0.6477 (3)	0.0462 (11)	
H17	0.3087	0.3453	0.6970	0.055*	
C18	0.3708 (6)	0.4873 (5)	0.6321 (4)	0.0636 (16)	
H18	0.4204	0.4983	0.6699	0.076*	
C19	0.3650 (6)	0.5618 (5)	0.5616 (4)	0.0606 (15)	
H19	0.4093	0.6255	0.5506	0.073*	
C20	0.2942 (5)	0.5436 (4)	0.5065 (4)	0.0505 (12)	
H20	0.2909	0.5940	0.4565	0.061*	
C21	0.2283 (4)	0.4512 (4)	0.5250 (3)	0.0383 (9)	
C22	0.1449 (4)	0.4264 (3)	0.4740 (3)	0.0364 (9)	
C23	0.1209 (5)	0.4921 (4)	0.4014 (3)	0.0464 (11)	
H23	0.1620	0.5577	0.3810	0.056*	
C24	0.0392 (5)	0.4629 (4)	0.3597 (3)	0.0494 (12)	
H24	0.0254	0.5066	0.3089	0.059*	
C25	−0.0240 (5)	0.3690 (4)	0.3916 (3)	0.0472 (12)	
H25	−0.0830	0.3478	0.3641	0.057*	
C26	0.0011 (5)	0.3071 (4)	0.4642 (3)	0.0405 (10)	
H26	−0.0428	0.2430	0.4870	0.049*	
C27	0.3784 (4)	0.2201 (4)	0.4883 (3)	0.0418 (10)	
H27	0.3987	0.2876	0.4929	0.050*	
C28	0.4621 (5)	0.1703 (4)	0.4364 (4)	0.0495 (12)	
H28	0.5392	0.2032	0.4054	0.059*	
C29	0.4349 (5)	0.0727 (4)	0.4290 (3)	0.0464 (11)	
H29	0.4916	0.0380	0.3919	0.056*	
C30	0.3234 (5)	0.0257 (4)	0.4765 (3)	0.0415 (10)	
H30	0.3037	−0.0428	0.4741	0.050*	
C31	0.2414 (4)	0.0790 (3)	0.5273 (3)	0.0329 (9)	
C32	0.1175 (4)	0.0400 (3)	0.5761 (3)	0.0337 (9)	
C33	0.0747 (5)	−0.0597 (4)	0.5847 (3)	0.0415 (10)	
H33	0.1283	−0.1095	0.5622	0.050*	
C34	−0.0470 (5)	−0.0855 (4)	0.6263 (3)	0.0437 (11)	
H34	−0.0774	−0.1542	0.6348	0.052*	
C35	−0.1245 (4)	−0.0112 (4)	0.6554 (3)	0.0410 (10)	
H35	−0.2102	−0.0267	0.6805	0.049*	
C36	−0.0763 (4)	0.0853 (4)	0.6478 (3)	0.0370 (9)	
H36	−0.1293	0.1360	0.6693	0.044*	
C37	0.3274 (11)	0.3094 (8)	0.9504 (7)	0.106 (3)	
H37A	0.3329	0.2505	0.9978	0.127*	
H37B	0.2392	0.3155	0.9460	0.127*	
C38	0.0773 (10)	0.5757 (8)	0.8701 (6)	0.104 (3)	
H38A	0.1078	0.5725	0.9228	0.125*	
H38B	0.1143	0.6386	0.8284	0.125*	
C39	0.4343 (9)	0.3132 (9)	0.2089 (7)	0.115 (3)	
H39A	0.5017	0.2796	0.2351	0.138*	
H39B	0.4738	0.3558	0.1524	0.138*	
B1	0.6282 (6)	0.2076 (5)	0.6370 (4)	0.0492 (13)	
B2	0.7686 (6)	0.1829 (5)	0.2559 (4)	0.0522 (14)	
H1	0.093 (5)	0.165 (4)	0.835 (4)	0.043*	

### Atomic displacement parameters (Å^2^)

**Table:**

	*U*^11^	*U*^22^	*U*^33^	*U*^12^	*U*^13^	*U*^23^
Ru1	0.0285 (2)	0.0341 (2)	0.03044 (19)	−0.00236 (14)	−0.00396 (14)	−0.00737 (14)
Cl1	0.0815 (15)	0.1179 (19)	0.158 (2)	−0.0026 (13)	−0.0007 (15)	−0.0754 (18)
Cl2	0.1014 (16)	0.0982 (15)	0.1238 (18)	0.0138 (13)	−0.0511 (15)	−0.0491 (13)
Cl3	0.098 (2)	0.242 (5)	0.222 (5)	0.018 (3)	−0.008 (3)	−0.103 (4)
Cl4	0.144 (3)	0.151 (3)	0.171 (3)	0.030 (2)	−0.078 (2)	−0.087 (2)
Cl5	0.161 (3)	0.189 (3)	0.132 (2)	0.082 (3)	−0.038 (2)	−0.073 (2)
Cl6	0.128 (2)	0.119 (2)	0.162 (3)	−0.0202 (19)	−0.008 (2)	−0.010 (2)
F1	0.058 (2)	0.112 (4)	0.116 (4)	0.025 (2)	−0.033 (3)	−0.021 (3)
F2	0.095 (3)	0.091 (3)	0.088 (3)	−0.012 (2)	−0.041 (3)	−0.034 (2)
F3	0.078 (3)	0.057 (2)	0.073 (2)	−0.0024 (19)	0.007 (2)	0.0039 (18)
F4	0.089 (3)	0.057 (2)	0.062 (2)	−0.0085 (19)	0.0078 (19)	−0.0097 (16)
F5	0.083 (3)	0.091 (3)	0.073 (2)	0.005 (2)	−0.037 (2)	−0.007 (2)
F6	0.151 (5)	0.073 (3)	0.086 (3)	−0.035 (3)	−0.056 (3)	−0.010 (2)
F7	0.063 (2)	0.070 (2)	0.059 (2)	−0.0186 (18)	0.0040 (17)	−0.0126 (17)
F8	0.053 (2)	0.157 (5)	0.080 (3)	0.013 (3)	0.012 (2)	−0.005 (3)
O1	0.0318 (15)	0.0429 (17)	0.0311 (14)	−0.0014 (13)	−0.0034 (12)	−0.0099 (12)
N1	0.036 (2)	0.049 (2)	0.0353 (18)	0.0017 (17)	−0.0058 (16)	−0.0083 (16)
N2	0.0312 (18)	0.039 (2)	0.0313 (17)	−0.0015 (15)	−0.0015 (15)	−0.0081 (15)
N3	0.036 (2)	0.048 (2)	0.0317 (18)	0.0078 (17)	−0.0050 (16)	−0.0075 (17)
N4	0.045 (2)	0.052 (2)	0.039 (2)	0.0017 (19)	−0.0054 (18)	−0.0112 (18)
N5	0.0287 (18)	0.0363 (19)	0.0356 (18)	0.0000 (15)	−0.0026 (15)	−0.0098 (15)
N6	0.0325 (19)	0.0349 (19)	0.0321 (17)	−0.0021 (15)	−0.0044 (15)	−0.0081 (14)
N7	0.0290 (18)	0.040 (2)	0.0335 (17)	−0.0039 (15)	−0.0030 (15)	−0.0107 (15)
N8	0.0304 (18)	0.0337 (18)	0.0292 (16)	0.0000 (14)	−0.0049 (14)	−0.0042 (14)
C1	0.038 (2)	0.043 (3)	0.039 (2)	−0.003 (2)	−0.008 (2)	−0.0024 (19)
C2	0.045 (3)	0.059 (3)	0.040 (2)	0.003 (2)	−0.005 (2)	−0.005 (2)
C3	0.055 (3)	0.068 (4)	0.040 (3)	−0.002 (3)	−0.006 (2)	−0.009 (2)
C4	0.055 (3)	0.069 (4)	0.036 (2)	−0.008 (3)	−0.015 (2)	0.001 (2)
C5	0.046 (3)	0.049 (3)	0.045 (3)	−0.003 (2)	−0.019 (2)	−0.002 (2)
C6	0.037 (2)	0.045 (3)	0.044 (2)	−0.007 (2)	−0.012 (2)	−0.004 (2)
C7	0.037 (2)	0.046 (3)	0.052 (3)	0.003 (2)	−0.014 (2)	−0.009 (2)
C8	0.037 (2)	0.048 (3)	0.039 (2)	0.001 (2)	−0.006 (2)	−0.009 (2)
C9	0.035 (2)	0.039 (2)	0.038 (2)	−0.0033 (19)	−0.0049 (19)	−0.0068 (18)
C10	0.030 (2)	0.041 (2)	0.036 (2)	−0.0007 (18)	−0.0043 (18)	−0.0088 (18)
C11	0.032 (2)	0.039 (2)	0.037 (2)	−0.0015 (18)	−0.0025 (18)	−0.0122 (18)
C12	0.034 (2)	0.043 (2)	0.037 (2)	−0.0055 (19)	−0.0028 (19)	−0.0114 (19)
C13	0.038 (3)	0.050 (3)	0.044 (2)	0.005 (2)	−0.004 (2)	−0.009 (2)
C14	0.042 (3)	0.061 (3)	0.053 (3)	0.008 (2)	−0.004 (2)	−0.018 (3)
C15	0.047 (3)	0.064 (3)	0.049 (3)	0.003 (3)	0.005 (2)	−0.021 (3)
C16	0.053 (3)	0.061 (3)	0.036 (2)	−0.002 (3)	−0.003 (2)	−0.014 (2)
C17	0.042 (3)	0.050 (3)	0.049 (3)	−0.004 (2)	−0.011 (2)	−0.014 (2)
C18	0.056 (3)	0.066 (4)	0.079 (4)	−0.017 (3)	−0.023 (3)	−0.025 (3)
C19	0.052 (3)	0.052 (3)	0.079 (4)	−0.019 (3)	−0.012 (3)	−0.016 (3)
C20	0.041 (3)	0.044 (3)	0.060 (3)	−0.010 (2)	−0.003 (2)	−0.003 (2)
C21	0.031 (2)	0.038 (2)	0.043 (2)	−0.0027 (18)	−0.0013 (19)	−0.0098 (19)
C22	0.033 (2)	0.036 (2)	0.036 (2)	0.0003 (18)	0.0014 (18)	−0.0081 (18)
C23	0.055 (3)	0.038 (2)	0.041 (2)	−0.002 (2)	−0.003 (2)	−0.0031 (19)
C24	0.055 (3)	0.046 (3)	0.043 (3)	0.005 (2)	−0.011 (2)	−0.002 (2)
C25	0.051 (3)	0.050 (3)	0.047 (3)	0.005 (2)	−0.020 (2)	−0.014 (2)
C26	0.041 (3)	0.041 (2)	0.040 (2)	−0.002 (2)	−0.010 (2)	−0.0094 (19)
C27	0.030 (2)	0.048 (3)	0.046 (2)	−0.006 (2)	−0.0010 (19)	−0.013 (2)
C28	0.032 (2)	0.058 (3)	0.055 (3)	−0.007 (2)	0.002 (2)	−0.016 (2)
C29	0.036 (2)	0.058 (3)	0.047 (3)	0.003 (2)	−0.002 (2)	−0.023 (2)
C30	0.038 (2)	0.044 (3)	0.045 (2)	0.001 (2)	−0.009 (2)	−0.015 (2)
C31	0.032 (2)	0.036 (2)	0.033 (2)	0.0001 (17)	−0.0099 (17)	−0.0092 (17)
C32	0.032 (2)	0.035 (2)	0.034 (2)	−0.0029 (18)	−0.0084 (17)	−0.0051 (16)
C33	0.042 (3)	0.040 (2)	0.044 (2)	0.000 (2)	−0.009 (2)	−0.0115 (19)
C34	0.045 (3)	0.038 (2)	0.045 (2)	−0.011 (2)	−0.007 (2)	−0.0057 (19)
C35	0.033 (2)	0.046 (3)	0.041 (2)	−0.009 (2)	−0.0024 (19)	−0.006 (2)
C36	0.030 (2)	0.043 (2)	0.035 (2)	−0.0026 (19)	−0.0031 (18)	−0.0073 (18)
C37	0.111 (8)	0.100 (7)	0.106 (7)	−0.020 (6)	−0.017 (6)	−0.026 (5)
C38	0.105 (7)	0.101 (6)	0.095 (6)	−0.039 (6)	0.006 (5)	−0.022 (5)
C39	0.070 (6)	0.151 (9)	0.121 (8)	0.012 (6)	0.004 (5)	−0.051 (7)
B1	0.043 (3)	0.054 (3)	0.050 (3)	−0.003 (3)	−0.009 (3)	−0.011 (3)
B2	0.045 (3)	0.059 (4)	0.051 (3)	−0.004 (3)	−0.011 (3)	−0.008 (3)

### Geometric parameters (Å, º)

**Table:**

Ru1—N7	2.036 (4)	C9—C10	1.461 (6)
Ru1—N2	2.047 (4)	C10—H10	0.9500
Ru1—N8	2.049 (4)	C11—C12	1.480 (6)
Ru1—N5	2.054 (4)	C12—C13	1.370 (7)
Ru1—N6	2.056 (4)	C13—C14	1.376 (7)
Ru1—O1	2.090 (3)	C13—H13	0.9500
Cl1—C37	1.770 (9)	C14—C15	1.377 (8)
Cl2—C37	1.755 (10)	C14—H14	0.9500
Cl3—C38	1.654 (11)	C15—C16	1.369 (8)
Cl4—C38	1.700 (10)	C15—H15	0.9500
Cl5—C39	1.688 (10)	C16—H16	0.9500
Cl6—C39	1.786 (11)	C17—C18	1.374 (8)
F1—B1	1.351 (8)	C17—H17	0.9500
F2—B1	1.371 (7)	C18—C19	1.365 (9)
F3—B1	1.369 (8)	C18—H18	0.9500
F4—B1	1.399 (7)	C19—C20	1.382 (8)
F5—B2	1.372 (7)	C19—H19	0.9500
F6—B2	1.371 (8)	C20—C21	1.383 (7)
F7—B2	1.370 (7)	C20—H20	0.9500
F8—B2	1.370 (8)	C21—C22	1.460 (6)
O1—C11	1.248 (5)	C22—C23	1.394 (7)
N1—C9	1.322 (6)	C23—C24	1.358 (8)
N1—C1	1.362 (6)	C23—H23	0.9500
N2—C10	1.287 (6)	C24—C25	1.385 (8)
N2—N3	1.386 (5)	C24—H24	0.9500
N3—C11	1.321 (6)	C25—C26	1.377 (7)
N3—H1	0.76 (6)	C25—H25	0.9500
N4—C16	1.330 (7)	C26—H26	0.9500
N4—C12	1.350 (6)	C27—C28	1.364 (7)
N5—C17	1.338 (6)	C27—H27	0.9500
N5—C21	1.367 (6)	C28—C29	1.373 (7)
N6—C26	1.337 (6)	C28—H28	0.9500
N6—C22	1.356 (6)	C29—C30	1.382 (7)
N7—C27	1.335 (6)	C29—H29	0.9500
N7—C31	1.368 (5)	C30—C31	1.372 (6)
N8—C36	1.345 (6)	C30—H30	0.9500
N8—C32	1.351 (6)	C31—C32	1.464 (6)
C1—C2	1.407 (7)	C32—C33	1.383 (6)
C1—C6	1.417 (7)	C33—C34	1.376 (7)
C2—C3	1.367 (7)	C33—H33	0.9500
C2—H2	0.9500	C34—C35	1.375 (7)
C3—C4	1.412 (8)	C34—H34	0.9500
C3—H3	0.9500	C35—C36	1.368 (6)
C4—C5	1.344 (8)	C35—H35	0.9500
C4—H4	0.9500	C36—H36	0.9500
C5—C6	1.416 (6)	C37—H37A	0.9900
C5—H5	0.9500	C37—H37B	0.9900
C6—C7	1.400 (7)	C38—H38A	0.9900
C7—C8	1.359 (7)	C38—H38B	0.9900
C7—H7	0.9500	C39—H39A	0.9900
C8—C9	1.409 (7)	C39—H39B	0.9900
C8—H8	0.9500		
			
N7—Ru1—N2	96.41 (15)	C19—C18—C17	119.4 (5)
N7—Ru1—N8	79.11 (14)	C19—C18—H18	120.3
N2—Ru1—N8	87.08 (14)	C17—C18—H18	120.3
N7—Ru1—N5	95.80 (14)	C18—C19—C20	119.4 (5)
N2—Ru1—N5	96.69 (14)	C18—C19—H19	120.3
N8—Ru1—N5	174.01 (13)	C20—C19—H19	120.3
N7—Ru1—N6	90.26 (15)	C19—C20—C21	119.2 (5)
N2—Ru1—N6	172.50 (14)	C19—C20—H20	120.4
N8—Ru1—N6	97.59 (14)	C21—C20—H20	120.4
N5—Ru1—N6	79.15 (14)	N5—C21—C20	121.0 (4)
N7—Ru1—O1	172.56 (13)	N5—C21—C22	115.1 (4)
N2—Ru1—O1	78.77 (13)	C20—C21—C22	123.8 (5)
N8—Ru1—O1	94.89 (13)	N6—C22—C23	120.0 (4)
N5—Ru1—O1	90.42 (13)	N6—C22—C21	115.2 (4)
N6—Ru1—O1	94.92 (13)	C23—C22—C21	124.8 (4)
C11—O1—Ru1	111.9 (3)	C24—C23—C22	120.3 (5)
C9—N1—C1	119.3 (4)	C24—C23—H23	119.9
C10—N2—N3	117.3 (4)	C22—C23—H23	119.9
C10—N2—Ru1	132.9 (3)	C23—C24—C25	119.6 (5)
N3—N2—Ru1	109.9 (3)	C23—C24—H24	120.2
C11—N3—N2	117.9 (4)	C25—C24—H24	120.2
C11—N3—H1	128 (4)	C26—C25—C24	118.2 (5)
N2—N3—H1	114 (4)	C26—C25—H25	120.9
C16—N4—C12	116.4 (5)	C24—C25—H25	120.9
C17—N5—C21	118.5 (4)	N6—C26—C25	122.7 (5)
C17—N5—Ru1	126.5 (3)	N6—C26—H26	118.6
C21—N5—Ru1	114.9 (3)	C25—C26—H26	118.6
C26—N6—C22	119.2 (4)	N7—C27—C28	122.1 (4)
C26—N6—Ru1	125.6 (3)	N7—C27—H27	118.9
C22—N6—Ru1	115.2 (3)	C28—C27—H27	118.9
C27—N7—C31	118.5 (4)	C27—C28—C29	119.9 (5)
C27—N7—Ru1	126.1 (3)	C27—C28—H28	120.0
C31—N7—Ru1	115.3 (3)	C29—C28—H28	120.0
C36—N8—C32	119.0 (4)	C28—C29—C30	118.8 (5)
C36—N8—Ru1	125.9 (3)	C28—C29—H29	120.6
C32—N8—Ru1	114.9 (3)	C30—C29—H29	120.6
N1—C1—C2	118.6 (5)	C31—C30—C29	119.3 (4)
N1—C1—C6	121.4 (4)	C31—C30—H30	120.4
C2—C1—C6	120.0 (4)	C29—C30—H30	120.4
C3—C2—C1	120.0 (5)	N7—C31—C30	121.3 (4)
C3—C2—H2	120.0	N7—C31—C32	114.4 (4)
C1—C2—H2	120.0	C30—C31—C32	124.2 (4)
C2—C3—C4	119.8 (5)	N8—C32—C33	121.2 (4)
C2—C3—H3	120.1	N8—C32—C31	114.8 (4)
C4—C3—H3	120.1	C33—C32—C31	124.0 (4)
C5—C4—C3	121.2 (5)	C34—C33—C32	118.9 (5)
C5—C4—H4	119.4	C34—C33—H33	120.6
C3—C4—H4	119.4	C32—C33—H33	120.6
C4—C5—C6	120.7 (5)	C35—C34—C33	119.6 (4)
C4—C5—H5	119.6	C35—C34—H34	120.2
C6—C5—H5	119.6	C33—C34—H34	120.2
C7—C6—C5	124.4 (5)	C36—C35—C34	119.0 (4)
C7—C6—C1	117.5 (4)	C36—C35—H35	120.5
C5—C6—C1	118.1 (5)	C34—C35—H35	120.5
C8—C7—C6	120.5 (5)	N8—C36—C35	122.0 (4)
C8—C7—H7	119.7	N8—C36—H36	119.0
C6—C7—H7	119.7	C35—C36—H36	119.0
C7—C8—C9	118.8 (4)	Cl2—C37—Cl1	111.0 (6)
C7—C8—H8	120.6	Cl2—C37—H37A	109.4
C9—C8—H8	120.6	Cl1—C37—H37A	109.4
N1—C9—C8	122.5 (4)	Cl2—C37—H37B	109.4
N1—C9—C10	118.1 (4)	Cl1—C37—H37B	109.4
C8—C9—C10	119.4 (4)	H37A—C37—H37B	108.0
N2—C10—C9	128.1 (4)	Cl3—C38—Cl4	113.1 (6)
N2—C10—H10	115.9	Cl3—C38—H38A	109.0
C9—C10—H10	115.9	Cl4—C38—H38A	109.0
O1—C11—N3	121.4 (4)	Cl3—C38—H38B	109.0
O1—C11—C12	122.6 (4)	Cl4—C38—H38B	109.0
N3—C11—C12	116.0 (4)	H38A—C38—H38B	107.8
N4—C12—C13	124.1 (4)	Cl5—C39—Cl6	114.6 (6)
N4—C12—C11	114.9 (4)	Cl5—C39—H39A	108.6
C13—C12—C11	121.0 (4)	Cl6—C39—H39A	108.6
C12—C13—C14	118.3 (5)	Cl5—C39—H39B	108.6
C12—C13—H13	120.9	Cl6—C39—H39B	108.6
C14—C13—H13	120.9	H39A—C39—H39B	107.6
C13—C14—C15	118.3 (5)	F1—B1—F3	113.2 (5)
C13—C14—H14	120.9	F1—B1—F2	111.3 (5)
C15—C14—H14	120.9	F3—B1—F2	109.4 (5)
C16—C15—C14	119.9 (5)	F1—B1—F4	108.2 (5)
C16—C15—H15	120.1	F3—B1—F4	108.2 (5)
C14—C15—H15	120.1	F2—B1—F4	106.3 (5)
N4—C16—C15	123.0 (5)	F8—B2—F7	108.7 (6)
N4—C16—H16	118.5	F8—B2—F6	110.9 (6)
C15—C16—H16	118.5	F7—B2—F6	108.2 (5)
N5—C17—C18	122.5 (5)	F8—B2—F5	108.1 (5)
N5—C17—H17	118.8	F7—B2—F5	110.7 (5)
C18—C17—H17	118.8	F6—B2—F5	110.3 (6)
			
C10—N2—N3—C11	179.3 (4)	C17—N5—C21—C20	−0.2 (7)
Ru1—N2—N3—C11	−1.9 (5)	Ru1—N5—C21—C20	−178.0 (4)
C9—N1—C1—C2	179.0 (5)	C17—N5—C21—C22	−177.6 (4)
C9—N1—C1—C6	0.8 (7)	Ru1—N5—C21—C22	4.7 (5)
N1—C1—C2—C3	−178.0 (5)	C19—C20—C21—N5	−0.8 (8)
C6—C1—C2—C3	0.2 (8)	C19—C20—C21—C22	176.3 (5)
C1—C2—C3—C4	−0.2 (9)	C26—N6—C22—C23	−1.2 (7)
C2—C3—C4—C5	−1.0 (9)	Ru1—N6—C22—C23	178.5 (4)
C3—C4—C5—C6	2.2 (8)	C26—N6—C22—C21	176.1 (4)
C4—C5—C6—C7	179.1 (5)	Ru1—N6—C22—C21	−4.2 (5)
C4—C5—C6—C1	−2.2 (7)	N5—C21—C22—N6	−0.3 (6)
N1—C1—C6—C7	−2.1 (7)	C20—C21—C22—N6	−177.6 (5)
C2—C1—C6—C7	179.8 (5)	N5—C21—C22—C23	176.8 (4)
N1—C1—C6—C5	179.1 (4)	C20—C21—C22—C23	−0.4 (8)
C2—C1—C6—C5	1.0 (7)	N6—C22—C23—C24	−1.2 (7)
C5—C6—C7—C8	−178.9 (5)	C21—C22—C23—C24	−178.2 (5)
C1—C6—C7—C8	2.3 (7)	C22—C23—C24—C25	2.5 (8)
C6—C7—C8—C9	−1.4 (7)	C23—C24—C25—C26	−1.5 (8)
C1—N1—C9—C8	0.3 (7)	C22—N6—C26—C25	2.3 (7)
C1—N1—C9—C10	−178.1 (4)	Ru1—N6—C26—C25	−177.4 (4)
C7—C8—C9—N1	0.0 (7)	C24—C25—C26—N6	−0.9 (8)
C7—C8—C9—C10	178.3 (4)	C31—N7—C27—C28	1.3 (7)
N3—N2—C10—C9	−1.0 (7)	Ru1—N7—C27—C28	−178.1 (4)
Ru1—N2—C10—C9	−179.4 (3)	N7—C27—C28—C29	−0.2 (8)
N1—C9—C10—N2	−0.4 (7)	C27—C28—C29—C30	−1.6 (8)
C8—C9—C10—N2	−178.8 (5)	C28—C29—C30—C31	2.3 (7)
Ru1—O1—C11—N3	1.5 (5)	C27—N7—C31—C30	−0.7 (6)
Ru1—O1—C11—C12	−179.3 (3)	Ru1—N7—C31—C30	178.8 (3)
N2—N3—C11—O1	0.3 (7)	C27—N7—C31—C32	−178.0 (4)
N2—N3—C11—C12	−179.0 (4)	Ru1—N7—C31—C32	1.6 (5)
C16—N4—C12—C13	−1.3 (7)	C29—C30—C31—N7	−1.1 (7)
C16—N4—C12—C11	179.7 (4)	C29—C30—C31—C32	175.9 (4)
O1—C11—C12—N4	−174.6 (4)	C36—N8—C32—C33	−5.6 (6)
N3—C11—C12—N4	4.6 (6)	Ru1—N8—C32—C33	169.3 (3)
O1—C11—C12—C13	6.4 (7)	C36—N8—C32—C31	172.1 (4)
N3—C11—C12—C13	−174.4 (5)	Ru1—N8—C32—C31	−12.9 (4)
N4—C12—C13—C14	1.8 (8)	N7—C31—C32—N8	7.5 (5)
C11—C12—C13—C14	−179.3 (5)	C30—C31—C32—N8	−169.7 (4)
C12—C13—C14—C15	−1.0 (8)	N7—C31—C32—C33	−174.8 (4)
C13—C14—C15—C16	−0.1 (9)	C30—C31—C32—C33	8.0 (7)
C12—N4—C16—C15	0.1 (8)	N8—C32—C33—C34	2.7 (7)
C14—C15—C16—N4	0.6 (9)	C31—C32—C33—C34	−174.8 (4)
C21—N5—C17—C18	0.6 (8)	C32—C33—C34—C35	2.4 (7)
Ru1—N5—C17—C18	178.0 (4)	C33—C34—C35—C36	−4.6 (7)
N5—C17—C18—C19	0.2 (9)	C32—N8—C36—C35	3.4 (6)
C17—C18—C19—C20	−1.2 (10)	Ru1—N8—C36—C35	−170.9 (3)
C18—C19—C20—C21	1.5 (9)	C34—C35—C36—N8	1.7 (7)

### Hydrogen-bond geometry (Å, º)

**Table:**

*D*—H···*A*	*D*—H	H···*A*	*D*···*A*	*D*—H···*A*
N3—H1···N1	0.76 (6)	1.90 (6)	2.553 (6)	145 (6)
